# Genetics of the Inverse Relationship between Grain Yield and Grain Protein Content in Common Wheat

**DOI:** 10.3390/plants11162146

**Published:** 2022-08-18

**Authors:** Manuel Geyer, Volker Mohler, Lorenz Hartl

**Affiliations:** Bavarian State Research Center for Agriculture, Institute for Crop Science and Plant Breeding, 85354 Freising, Germany

**Keywords:** grain protein deviation, grain yield deviation, MAGIC, protein yield, QTL

## Abstract

Grain protein content (GPC) is one of the most important criteria to determine the quality of common wheat (*Triticum aestivum*). One of the major obstacles for bread wheat production is the negative correlation between GPC and grain yield (GY). Previous studies demonstrated that the deviation from this inverse relationship is highly heritable. However, little is known about the genetics controlling these deviations in common wheat. To fill this gap, we performed quantitative trait locus (QTL) analysis for GY, GPC, and four derived GY-GPC indices using an eight-way multiparent advanced generation intercross population comprising 394 lines. Interval mapping was conducted using phenotypic data from up to nine environments and genotypic data from a 20k single-nucleotide polymorphism array. The four indices were highly heritable (0.76–0.88) and showed distinct correlations to GY and GPC. Interval mapping revealed that GY, GPC, and GY-GPC indices were controlled by 6, 12, and 12 unique QTL, of which each explained only a small amount of phenotypic variance (*R*^2^ ≤ 10%). Ten of the 12 index QTL were independent of loci affecting GY and GPC. QTL regions harboured several candidate genes, including *Rht-1*, *WAPO-A1*, *TaTEF-7A*, and *NRT2.6-7A*. The study confirmed the usefulness of indices to mitigate the inverse GY-GPC relationship in breeding, though the selection method should reflect their polygenic inheritance.

## 1. Introduction

Wheat is the most widely grown crop worldwide, occupying 15% of arable land, with common wheat (*Triticum aestivum*) being the most important species. As wheat provides 18% of consumed calories and 20% of protein intake, it is of critical importance for global food safety [1]. Wheat production has surged in the last few decades, and further production growth is necessary to meet future demands [2]. Boosting grain yield (GY) through genetic improvement of cultivars has been highlighted as one of the most promising solutions to reach this goal [3]. Wheat quality, which is associated with processing attributes, is another important criterion for breeding wheat cultivars. The end-use of wheat is determined by the grain protein content (GPC) and the composition of the proteins. Wheat grains usually contain about 7–17% protein, of which about 80% belongs to the gluten storage proteins glutenin and gliadin. The quantity, ratio, and allelic variants of these gluten proteins are essential determinants for baking quality, as they affect the viscoelastic properties of the dough and the final loaf volume. Other proteins important for wheat quality include puroindolines, which affect grain hardness and, thereby, the milling and baking properties, and enzymes such as starch-degrading α-amylase, which can result in poor product quality. Although the composition of proteins is important for wheat quality, the overall GPC is one of the most important criteria to determine the end-use of wheat grains. Generally, wheat with a moderate-to-high GPC (>12%) is necessary for pan breads, whereas wheat with a lower GPC is usually used for cookies, noodles, or animal feed [4]. GPC is influenced by many factors, including genetics, the availability of water and nitrogen, heat stress, and the length of the grain-filling period [5,6]. One of the major obstacles for the production of baking wheat is the well-documented inverse relationship between GPC and GY. Oury and Godin [7] and Laidig et al. [8] investigated GY and GPC in winter wheat variety trials in France and Germany across 11 and 32 years and found mean correlations of −0.45 and −0.77, respectively. The large difference between the two correlation coefficients indicates that environmental and genetic factors could influence the relationship between the two traits. Indeed, it was demonstrated that the deviation from the linear regression of GPC on GY, which was termed grain protein deviation (GPD), is a heritable trait [9]. Several follow-up studies about possible physiological processes conditioning GPD concluded that post-anthesis nitrogen uptake is the most crucial factor, whereas remobilization of nitrogen within the plant is less important [10,11,12]. Consequently, GPD and several other indices derived from GY and GPC were suggested as selection criteria to mitigate the negative relationship between the two traits in future wheat varieties. Genetic studies to identify quantitative trait loci (QTL) affecting GPD and related indices were conducted in durum wheat (*Triticum turgidum*) [13,14], hybrid wheat [15], and triticale (*×Triticosecale*) [16]. In common wheat, several studies analysed the genetic basis of GY and GPC to distinguish antagonistic QTL controlling both traits from QTL affecting only one of them [17,18,19]. However, apart from an initial gene expression study [20], nothing is known about the genetic architecture of GY-GPC indices in inbred lines of common wheat.

To fill this gap, we performed QTL analysis using the eight-way multiparent advanced generation intercross (MAGIC) population BMWpop, which captures a large part of the allelic diversity of the German wheat gene pool [21]. The studied traits included GY, GPC, and the derived indices GPD, grain yield deviation (GYD), grain protein yield (GPY), and an index with equal weights for GY and GPC (EWPY). To identify possible pleiotropic effects, the BMWpop was also analysed for heading date (HD), plant height (PH), thousand-grain weight (TGW), grain width (GW), and grain length (GL). The aims of our study were to (1) estimate the heritability and correlations of the GY-GPC indices, (2) investigate their genetic architecture, and (3) identify putative candidate genes underlying these traits.

## 2. Results

### 2.1. Phenotypic Analysis

Heritability estimates in the BMWpop were moderate to high, with 0.83 for GY and 0.93 for GPC. For the derived traits GPD, GYD, GPY, and EWPY, we estimated heritabilities between 0.76 and 0.88. Genotypic effects were significant for all traits, whereas environmental effects were significant for all traits except for the indices GPD, GYD, and EWPY, which have, by definition, an expected environmental effect of zero. The BMWpop had an average GY of 8.1 t/ha and a mean GPC of 13.6% (Table 1). The mean of the BMWpop was not significantly different from the mean of the founders for any of the traits. Ambition was the founder with the highest GY (9.2 t/ha), GYD (0.7 t/ha), and GPY (1.2 t/ha). Two, one, and 32 descendants exhibited higher phenotypic values for these traits, respectively. The highest GPC (14.6%) and GPD (0.5%) among the eight founders was observed for Bussard, which was surpassed by 29 and 65 lines, respectively. A total of 49 descendants showed higher EWPY than the best founder Format (0.5). The distribution of the mean trait values of the BMWpop is illustrated in Figure S1.

As expected, GY was inversely correlated with GPC across environments (*r* = −0.60). Within the eight trials, correlation ranged between −0.37 and −0.76. The derived index GPD was strongly associated with GPC (*r* = 0.92), whereas GYD (*r* = 0.86) was highly correlated with GY. The indices GPY and EWPY showed less pronounced correlations with GY and GPC (0.24 ≤ *r* ≤ 0.58). Late heading was negatively associated with GY and all indices (−0.20 ≤ *r* ≤ −0.10), whereas no relation to GPC was observed. A tall plant stature was accompanied by higher values for GPC, GPD, GPY, and EWPY (0.14 ≤ *r* ≤ 0.25). Large grains were positively associated with GY, GPC, and all indices (0.13 ≤ *r* ≤ 0.39; Table 2).

### 2.2. QTL Analysis

#### 2.2.1. Summary

**Figure 1:**
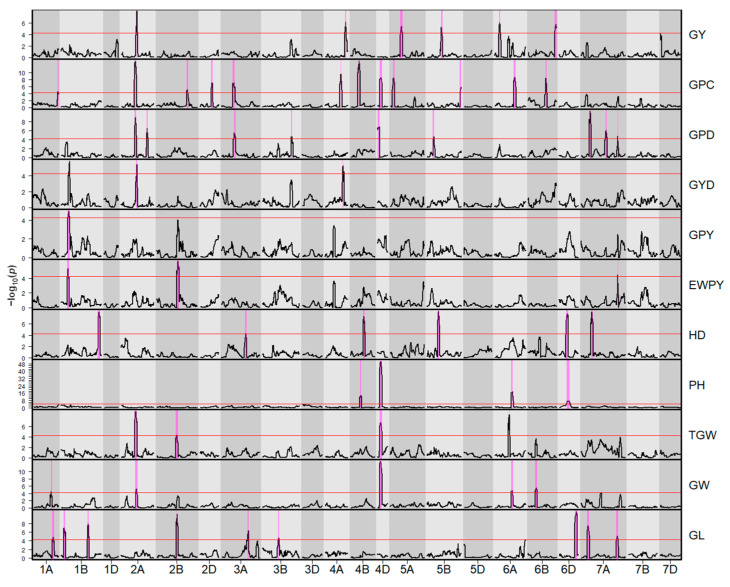
*p*-value curves for quantitative trait loci detected across environments: grain yield (GY), grain protein content (GPC), grain protein deviation (GPD), grain yield deviation (GYD), grain protein yield (GPY), equal-weight protein yield (EWPY), heading date (HD), plant height (PH), thousand-grain weight (TGW), grain width (GW), and grain length (GL). The significance threshold is represented by the red line. Support intervals are drawn as magenta stripes.

Composite interval mapping resulted in 62 QTL that were significant across environments. These QTL were mapped to 44 unique genomic regions. The number of QTL for a specific trait ranged between one for GPY and 12 for GPC. The highest *R*^2^ value within a trait varied between 1% for EWPY and 27% for PH. The most important QTL are characterized in Figure 1, Table 3, and the following subsections. Detailed results of QTL analysis including environment-specific QTL and founder effects are listed in Table S1. A summary of high-confidence gene models for detected QTL is shown in Table S2.

#### 2.2.2. Grain Yield

Interval mapping revealed six QTL for GY on chromosomes 2A, 4A, 5A, 5B, 6A, and 6B. Four of these QTL were detected in up to three of nine environments, whereas two of them were only detected across trials. The proportion of explained phenotypic variance across environments ranged between 4% and 10%. The highest *R*^2^ was observed for *QGy.lfl-2A*, which was detected in three trials. Lines that shared the lowest and highest yielding haplotypes at this locus differed by 0.7 t/ha. Interestingly, the most significant markers for another QTL, namely *QGy.lfl-4A,* were located 1.2 megabases (Mb) proximal to a homolog of *GRAIN SIZE 3* (*TaGS3-4A*) from rice (*Oryza* spp.). This locus explained 8% of the GY variance and was detected in three trials. The two most contrasting haplotypes differed by 0.6 t/ha.

#### 2.2.3. Grain Protein Content

GPC was controlled by 12 QTL on chromosomes 1A, 2A, 2B, 2D, 3A, 4A, 4B, 4D, 5A, 5B, 6A, and 6B. These QTL were found in one to six of the nine evaluated trials except for two QTL, which were only detected across trials. The amount of explained phenotypic variance ranged between <1% and 10%. The largest proportion of variance was explained by *QGpc.lfl-4B*, which was detected in three environments. This locus was mapped to the marker *TG0010*, which is a gene-derived marker for the reduced height gene *Rht-B1*. Using this marker, we found that the height-reducing allele *Rht-B1b* was associated with a GPC decrease of 0.54 percentage points. Besides this candidate gene, we found the glutamine synthetase *TaGSe-4B* 3.9 Mb proximal to *Rht-B1*. Another candidate gene associated with GPC was the semi-dwarfing gene *Rht-D1*, represented by *QGpc.lfl-4D* with the gene-derived marker *TG0011*. *Rht-D1* was detected in four environments and explained 4% of phenotypic variance, and the height-reducing allele *Rht-D1b* reduced GPC by 0.43 percentage points.

#### 2.2.4. Indices Derived from Grain Yield and Grain Protein Content

For GPD, we detected nine QTL on chromosomes 2A, 3A, 3B, 4D, 5B, and 7A. These loci explained between <1% and 8% of the GPD variance and exhibited significant effects in one or two of eight trials, with two QTL being found only across environments. Seven of these QTL were independent of the QTL for GY and GPC. The highest amount of phenotypic variation was contributed by *QGpd.lfl-2A.1*, which was found in two environments. The two haplotypes with the lowest and highest GPD differed by 0.5 percentage points. On chromosome 7A, we detected three QTL for GPD in the vicinity of candidate genes. *QGpd.lfl-7A.1* was found to be located 1.4 Mb downstream of the transcription elongation factor gene *TaTEF-7A*. This QTL explained 4% of the phenotypic variance, was detected in one environment, and showed a maximum effect of 0.5 percentage points. The second QTL on this chromosome, *QGpd.lfl-7A.2*, was located near the nitrate transporter gene *NRT2.6-7A*. Four of the most significant markers for this locus were located 3.8–13.6 Mb distal to the candidate gene, whereas one peak marker on the genetic map was mapped 280.9 Mb proximal on the physical map. The effect of *QGpd.lfl-7A.2* was observed in one environment and explained 5% of the phenotypic variance. The most extreme haplotypes differed by 0.3 percentage points. The third QTL on chromosome 7A, *QGpd.lfl-7A.3*, was mapped 1.3 Mb distal to the candidate gene *WHEAT ORTHOLOG OF APO1* (*WAPO-A1*). This locus was observed in two environments and explained 4% of the GPD variance. Based on the findings of a preceding study [22], we assigned the eight founder haplotypes to two alleles and estimated that *WAPO-A1b* decreased GPD by 0.13 percentage points compared to *WAPO-A1a*.

QTL mapping for GYD resulted in three QTL on chromosomes 1B, 2A, and 4A. They contributed between 6% and 8% to the phenotypic variance and were observed in one and two of eight separate environments, with one QTL being detected only across environments. Two of these QTL were independent of the QTL for GY and GPC. The largest part of the GYD variance was contributed by *QGyd.lfl-1B*, which was observed in two trials and exhibited a maximum effect of 0.1 t/ha. One of the three QTL, *QGyd.lfl-4A*, was mapped 3.8-8.7 Mb proximal to a candidate gene encoding a sucrose synthase (TraesCS4A02G446700). This locus explained 8% of the GYD variance, was observed in one environment, and exhibited a maximum effect of 0.3 t/ha.

For GPY, we detected one QTL on chromosome 1B. *QGpy.lfl-1B* explained 8% of the observed variation and was detected in two environments. The haplotypes associated with the lowest and highest GPY differed by <0.1 t/ha. *QGpy.lfl-1B* did not show significant effects on GY or GPC, which this index was derived from.

EWPY was controlled by three QTL on chromosomes 1B, 2B, and 7A. These loci were detected in one environment or only across environments and contributed between 1% and 7% to the total EWPY variance. All three QTL were independent of the QTL for GY and GPC. The largest proportion of EWPY variation was explained by *QEwpy.lfl-2B*, which was found in one environment and showed a maximum effect of 0.4. Another QTL on chromosome 7A (*QEwpy.lfl-7A*) was mapped 1.3 Mb distal to *WAPO-A1*. *QEwpy.lfl-7A* was only found across environments and explained 1% of the EWPY variance. *WAPO-A1b* reduced EWPY by 0.14 compared to *WAPO-A1a*.

#### 2.2.5. Heading Date and Plant Height

QTL analysis for HD revealed six QTL on chromosomes 1B, 3A, 4B, 5B, 6D, and 7A, which explained between 2% and 10% of the phenotypic variance and were observed in one to three of seven environments. None of these QTL colocalized with the QTL for GY, GPC, and the traits derived thereof.

PH was controlled by QTL on chromosomes 4B, 4D, 6A, and 6D. These loci explained 3–27% of the phenotypic variance and could be detected in either six or all seven environments. The largest part of PH variation could be explained by *QPh.lfl-4D*, which was mapped to the gene-derived marker *TG0011* and, thus, could be identified as the semi-dwarfing gene *Rht-D1*. *Rht-D1* was found in all seven trials. Using the diagnostic marker, we estimated an effect of 10.4 cm. Although *QPh.lfl-4B* was mapped 32.1 Mb downstream of the semi-dwarfing gene *Rht-B1* and its marker *TG0010*, this gene is the most likely candidate. *Rht-B1* explained 11% of the PH variance and was found to be significant in six trials, and based on marker *TG0010*, we estimated an effect of 6.1 cm. We could not find a candidate gene in the vicinity of the peak marker for *QPh.lfl-6A*. However, a neighbouring marker on the genetic map (0.5 centimorgan distal) was located 2.6 Mb upstream of the reduced height gene *Rht24*, which most likely corresponds to this QTL. *QPh.lfl-6A* contributed 12% to PH variation, was found in all seven trials, and showed a maximum haplotype effect of 8.8 cm. Besides these previously described genes, we additionally detected a QTL on chromosome 6D (*QPh.lfl-6D*) in six trials. This locus explained 3% of phenotypic variation and affected PH by up to 4.2 cm.

#### 2.2.6. Grain Morphometric Traits

QTL analysis of TGW, GW, and GL detected a total of 18 QTL on chromosomes 1A, 1B, 2A, 2B, 3A, 3B, 4D, 6A, 6B, 6D, and 7A. These loci were detected in two to six environments, and the *R*^2^ ranged between <1% and 20%. The two QTL *QTgw.lfl-4D* and *QGw.lfl-4D* on chromosome 4D were found to explain the largest part of the phenotypic variance for TGW (13%) and GW (20%). The two QTL were detected in five of eight and six of seven environments and could be mapped to the gene-derived marker for *Rht-D1*. Using this marker, we found that allele *Rht-B1b* reduced TGW and GW by 2.7 g and 0.1 mm. The most important locus for GL was *QGl.lfl-1B.1*, which was found to be significant in six of seven environments, explained 11% of the phenotypic variance, and showed a maximum effect of 0.3 mm between founder haplotypes. *QGl.lfl-7A.2* could be assigned to *WAPO-A1*, which was located 1.2 Mb proximal. *QGl.lfl-7A.2* was detected in three environments and explained <1% of GL. *WAPO-A1b* reduced GL by 0.03 mm compared to *WAPO-A1a*.

#### 2.2.7. Coinciding QTL

Overall, we identified ten genomic regions that affected more than one trait, of which the following loci involved QTL for GY, GPC, or any of the derived indices. Chromosome 1B harboured a region with coinciding QTL for three indices: *QGyd.lfl-1B*, *QGpy.lfl-1B*, and *QEwpy.lfl-1B*. The direction of the founder effects was in line with the high positive correlation among the three traits. The haplotype of Ambition was always associated with the lowest value, whereas the haplotypes of FIRL3565 and Format were the most favourable. On chromosome 2A, we found a QTL cluster controlling six traits including GY (*QGy.lfl-2A*), GPC (*QGpc.lfl-2A*), indices (*QGpd.lfl-2A.1* and *QGyd.lfl-2A*), and grain morphometric traits (*QTgw.lfl-2A* and *QGw.lfl-2A*). The effect directions of this locus reflected the inverse relationship between GY vs. GPC and derived indices. The haplotype of Event resulted in the lowest value for GY, GYD, TGW, and GW. The same haplotype was associated with the highest value for GPC and the second-highest value for GPD. A genomic region on chromosome 2B affected the index EWPY and two grain morphometric traits. Although effect directions were not always conclusive, the haplotype with the highest TGW and GW (BAYP4535) was accompanied by above-average EWPY values, and the haplotype of Bussard showed the lowest values for all three traits. The effects of the two colocalized QTL for GPC and GPD on chromosome 3A followed the same direction across all eight founders, with the haplotypes of BAYP4535 and Format yielding the lowest and highest values for both traits. *QPh.lfl-4D* (*Rht-D1*) coincided with the QTL for GPC, TGW, and GW. The height-reducing allele *Rht-D1b* was always associated with lower GPC, TGW, and GW, indicating that this gene contributes to the positive correlation among these traits. The same observation was made for *QPh.lfl-6A* (*Rht24*) and its effect on GW. Although *QPh.lfl-4B* (*Rht-B1*) did not overlap with any other QTL, we mapped *QGpc.lfl-4B* to the gene-derived marker for *Rht-B1*. Analogous to the effects of *Rht-D1*, the height-reducing *Rht-B1b* allele from BAYP4535 was accompanied by a low GPC. *QPh.lfl-6D* was the only PH QTL not affecting GY parameters, GPC, or derived indices. Instead, the height-reducing haplotype of this QTL was associated with early heading. On chromosome 7A, we found three coinciding QTL for GPD, EWPY, and GL and suggested *WAPO-A1* as the causative gene. Assigning the eight haplotypes to the two *WAPO-A1* alleles showed that *WAPO-A1b* decreased GPD, EWPY, and GL compared to *WAPO-A1a.*

## 3. Discussion

### 3.1. Candidate Genes for GY, GPC, and Derived Indices

#### 3.1.1. *Rht-B1*, *Rht-D1*, and *Rht24*

The present study detected four loci for PH, of which three could be identified as *Rht-B1*, *Rht-D1*, and *Rht24*. The two homologs *Rht-B1* and *Rht-D1* on chromosomes 4B and 4D are the two most effective dwarfing genes in the worldwide wheat gene pool. They encode DELLA proteins that repress sensitivity to gibberellins, and the mutated alleles *Rht-B1b* and *Rht-D1b* are thought to produce more effective repressors, leading to a decreased stem elongation [23]. Besides several other pleiotropic effects, *Rht-1* mutants were reported to increase grain set and the number of tillers per area, whereas grain size/weight and GPC are known to be negatively affected. The effect of these alleles on GY depends on the genetic background and environmental conditions [24,25,26,27]. The present study confirmed the reported effects of *Rht-1* on grain morphometric traits (*Rht-D1*) and GPC (*Rht-B1* and *Rht-D1*). This was also reflected by the positive correlation among these traits. However, this observation did not manifest a significant effect of *Rht-1* on GY or any of the indices derived from GY and GPC. These contradictory findings can be explained by both (1) a non-significant increase in GY and (2) a deviation from the inverse GY-GPC relationship, which was also below the threshold for significance. The high heritability of GPC compared to GY and derived indices may also support this explanation. *Rht24* on chromosome 6A is a globally distributed height-reducing gene, which was recently found to encode a gibberellin 2-oxidase [28,29,30]. The allele *Rht24b* confers a higher gene expression and, thereby, leads to a reduced plant height. An initial study on the pleiotropic effects of *Rht24* in near-isogenic lines showed no impact on GY or any of its components [30]. Contrary to these previous findings, *Rht24b* was shown to decrease not only PH, but also GW in the present study. This suggests that, although *Rht-1* and *Rht24* play different physiological roles in the regulation of PH, these genes may have similar effects on yield-related traits. Taken together, we confirmed the pleiotropic effects of *Rht* genes on grain morphometric traits (*Rht-D1*, *Rht24*) and GPC (*Rht-1*), but neither of these genes contributed to a significant deviation from the inverse GY-GPC relationship.

#### 3.1.2. *WAPO-A1*

On chromosome 7A, we found the genomic region harbouring *WAPO-A1* to control GPD, EWPY, and GL. *WAPO-A1*, also known as *TaAPO-A1*, is an ortholog of *APO-1*, which encodes an F-box protein, which is a component of a ubiquitin ligase and is known to affect panicle development and spikelet number in rice [31]. Three independent QTL mapping studies suggested that *WAPO-A1* is a major causative gene for the variation of spikelet number per spike in common wheat [32,33,34]. Using mutants and transgenic plants, this hypothesis was recently confirmed [35]. There are three haplotypes, H1–H3, which correspond to the alleles *WAPO-A1a*, *WAPO-A1b*, and *WAPO-A1c-d*, respectively. In common wheat, *WAPO-A1a* and *WAPO-A1b* are by far the most frequent alleles, and *WAPO-A1b* is associated with the highest number of spikelets per spike, followed by *WAPO-A1c-d* and *WAPO-A1a* [32,35]. Studies on the effect of *WAPO-A1* on GY are still inconclusive. Whereas Muqaddasi et al. [33] found a slight negative effect of *WAPO-A1b* on GY in European varieties (mainly winter wheat), Kuzay et al. [32], Voss-Fels et al. [34], and Kuzay et al. [35] found positive effects in the genetic background of spring wheat. The effect on grain morphometric traits is also unclear [32]. In a preceding study exploiting the BMWpop, *WAPO-A1* was already suggested as a candidate gene for spikelet number using phenotypic data collected from two trials in the United Kingdom and one trial in Germany. Sequencing of parental alleles showed that the BMWpop segregated for *WAPO-A1a* and *WAPO-A1b* and that the latter increased the number of (fertile) spikelets per spike and spike length [22]. In the present study, *WAPO-A1b* was associated with a decreased GPD, EWPY, and GL, indicating that the benefit of an increased grain number per spike might be offset by unfavourable effects on GY and GPC. Indeed, *WAPO-A1b* was associated with a non-significant negative effect on both GY and GPC (data not shown). Together with the findings from Muqaddasi et al. [33], the results of the present study suggest that the effect of *WAPO-A1b* on spikelet number cannot be exploited to boost GY in all environments and genetic backgrounds. Especially wheat breeding programs aiming for high GPC at below-average GY penalty could benefit from *WAPO-A1a* instead.

#### 3.1.3. *TaGS3-4A*

Chromosome 4A was found to harbour a QTL for GY (*QGy.lfl-4A*) near *TaGS3-4A*. This candidate gene is a homolog of *OsGS3*, which is known to control the yield components grain weight, GW, GL, and grain thickness in rice [36]. Similar effects were observed in common wheat by Zhang et al. [37], who demonstrated that *TaGS3-4A* is a negative regulator of TGW and GL. However, effects of *TaGS3-4A* on GY have not been investigated yet, and in the present study, *QGy.lfl-4A* did not colocalize with loci for grain morphometric traits. Thus, we concluded that *TaGS3-4A* is not a strong candidate for *QGy.lfl-4A*, and further research is necessary to study the effect of *TaGS3-4A* and neighbouring genes on GY.

#### 3.1.4. *TaGSe-4B*

The locus *QGpc.lfl-4B* affecting GPC was mapped to *Rht-B1*, which is located 3.9 Mb distal from the glutamine synthetase *TaGSe-4B* on chromosome 4B. Glutamine synthetase is important for the assimilation of nitrogen by incorporating ammonium into glutamate. *TaGSe-4B* belongs to *GSe*, which is one of four glutamine synthetase families that can be separated according to a phylogenetic study in wheat [38]. Glutamine synthetase genes in wheat were associated with multiple GY-related traits, nitrogen uptake, and GPC [39,40,41,42]. Due to the proximity of *Rht-B1* and *TaGSe-4B*, the mapping resolution of the present study does not allow a definite conclusion on the causative gene for this QTL. However, since both *Rht-1* genes are known to affect GPC across many germplasm sources and geographic regions [24] and the interval is too large for complete linkage disequilibrium in the wheat gene pool, we assumed that *Rht-B1* is the more likely a candidate gene for *QGpc.lfl-4B*. The effect of *TaGSe-4B* and *Rht-B1* on GPC could be investigated in future studies by genome editing.

#### 3.1.5. *TaTEF-7A*

One of three QTL on chromosome 7A controlling GPD (*QGpd.lfl-7A.1*) was found in the vicinity of the transcription elongation factor *TaTEF-7A*. This candidate gene was found to affect grain number per spike in a Chinese wheat mini core collection and near-isogenic lines. Overexpression of *TaTEF-7A* in *Arabidopsis thaliana* enhanced grain length, silique number, and silique length. There are three haplotypes differing in polymorphisms in the promoter region, of which *Hap-7A-3* leads to the highest gene expression and most favourable effect on grain number per spike [43]. Interestingly, the same haplotype was also associated with an increased GL in a panel of mostly European winter wheat cultivars [44]. However, no effect on GPD or GPC has been shown so far. Further work is required to characterize the effects of the three haplotypes on GPD.

#### 3.1.6. *TaNRT2.6-7A*

Besides *TaTEF-7A* and *WAPO-A1*, we found a third candidate gene for GPD, *TaNRT2.6-7A*, on chromosome 7A (*QGpd.lfl-7A.2*). The nitrate transporter gene *TaNRT2.6-7A* is part of the *NRT2* gene family belonging to the high-affinity transport system, which predominates under a low nitrate concentration [45]. Phylogenetic analysis of *NRT2* genes across species revealed that the three homoeologous *TaNRT2.6* copies shared the highest similarity with *AtNRT2.7* from *Arabidopsis thaliana* and *OsNRT2.4* from rice. *TaNRT2.6* is mainly expressed in the leaves at all growth stages, whereas all other wheat *NRT2* genes were mainly expressed in the roots [46], indicating that *TaNRT2.6* may be important for nitrate transport from the leaves to the spike. However, *TaNRT2.6-7A* has not been functionally characterized, and its effect on agronomic traits is still unknown. Interestingly, a member of the same gene family, *NRT2-6A*, was shown to be significantly associated with GPD in durum wheat [14]. Furthermore, the two nitrate transporters *NRT2.1* and *TaNRT2.5* were both shown to control post-anthesis nitrogen uptake, which is associated with deviations from the inverse relationship between GY and GPC [47,48]. These findings indicate that *TaNRT2.6-7A* and other members of the *NRT2* family are important targets for future studies on indices derived from GY and GPC.

#### 3.1.7. Sucrose Synthase (TraesCS4A02G446700)

On chromosome 4A, we found a QTL for GYD (*QGyd.lfl-4A*) near the sucrose synthase TraesCS4A02G446700. Sucrose synthase catalyses the conversion from sucrose to UDP-glucose and fructose, which is the first step in the sucrose–starch conversion pathway. In a recent study, TraesCS4A02G446700 was found to be significantly associated with spike-layer uniformity, which is an important factor influencing GY [49]. Although this gene has not been characterized further, other wheat sucrose synthase genes, such as the two homoeologous series *TaSus1* and *TaSus2*, have been investigated in more detail. The effect of these genes on TGW has been demonstrated in several studies [50,51,52,53]. Whereas sucrose synthase has a major impact on TGW, no associations have been found with GY or derived indices so far.

#### 3.1.8. Limitations of Identified Candidate Genes

The identification of the abovementioned candidate genes is a crucial first step, which could be followed by validation, allele screening, functional characterization, and possibly, modification by genome editing. As a consequence of the limited mapping resolution, we searched for candidate genes in relatively large intervals of at least 10 Mb, which contained an average of 149 gene models. Thus, we focused on genes and gene families that were shown to affect relevant traits in previous studies. Since this procedure involves a high level of uncertainty, the identified candidate genes should be validated carefully by fine-mapping.

### 3.2. Colocalizing QTL from Previous Studies

Comparing the detected QTL with those from previous studies using physical positions of support intervals revealed that 11 of 62 QTL overlapped with previously reported loci. For GY, HD, PH, and grain morphometric traits, the QTL *QGy.lfl-6A*, *QHd.lfl-4B*, *QPh.lfl-4B*, *QTgw.lfl-2A*, *QGw.lfl-2A*, and *QGl.lfl-1B.1* were mapped to meta-QTL for the respective traits [54]. Three of the original QTL used for a meta-QTL analysis of GPC [55] were found to overlap with the following QTL from the present study: *QGpc.lfl-2A*, *QGpc.lfl-3A*, and *QGpc.lfl-4B*. Furthermore, two of the marker–trait associations for GPD in hybrid wheat and durum wheat could be confirmed (*QGpd.lfl-3A* and *QGpd.lfl-5B*), whereas no coinciding QTL were found for other indices [14,15]. The observation that only 11 loci were mapped to reported QTL regions could be explained by the fact that many markers for previously identified QTL are not annotated in the reference genome sequence. Indeed, only 33% and 34% of the QTL reported in the meta-analyses by Saini et al. [54] and Gudi et al. [55] were anchored to the reference sequence of Chinese Spring using published marker annotations.

### 3.3. Implications for Quality Wheat Breeding

All indices derived from GY and GPC had a significant genetic variance and a high heritability, confirming that they are suitable for selection in wheat breeding, as suggested by previous studies [7,9,10,56]. The importance of these indices for selection is further substantiated by the fact that ten of the 12 unique index QTL were exclusively detected for derived indices, whereas only two of these QTL colocalized with the QTL for GY or GPC. The choice of the optimal index for selection depends on the focus of the breeding program. The observed correlations suggest that selection for high GPD will result in genotypes with a focus on high GPC and a below-average GY penalty. However, selection for this index will not exclude genotypes with low GY. In contrast, selection for high GYD will favour breeding lines with high GY at above-average GPC. Analogous to GPD, the selection for high GYD does not discard genotypes with low GPC. Using GPY as a criterion will result in a less unidirectional selection, though this index still favours a high GY versus high GPC. The index EWPY also showed balanced correlations to the original traits, allowing simultaneous improvement of both GY and GPC. As suggested by Neuweiler et al. [16], this index could be modified by adjusting the weights for these traits to the specific requirements of the breeding program. All indices revealed a polygenic inheritance with many QTL explaining only a small or medium amount of phenotypic variance. These findings indicate that marker-assisted selection is not suitable for the selection of favourable QTL alleles in elite breeding programs. Instead, we recommend phenotypic selection, genomic selection, or, as suggested by Michel et al. [57], genomics-assisted selection to counteract the inverse GY-GPC relationship. For quality wheat breeding, it is important to consider that essential quality traits such as loaf volume not only depend on GPC, but also on protein composition: it has been shown that the GPC of German winter wheat cultivars decreased significantly during 1983–2014, while at the same time, important quality traits were improved by breeding [8]. The composition of proteins is likely to gain importance, since nitrogen fertilization will be reduced due to economic and ecological reasons. Thus, future wheat breeding should follow a strategy combining both an improved protein quality and a high deviation from the inverse GY-GPC relationship.

## 4. Materials and Methods

### 4.1. Plant Material

QTL analysis was performed in an eight-way MAGIC population termed the BMWpop. This population comprises 394 F_6:8_ recombinant inbred lines derived from the Central European winter wheat lines Event, BAYP4535, Ambition, FIRL3565, Format, Potenzial, Bussard, and Julius, which cover all four quality groups as defined by the German Federal Plant Variety Office. The BMWpop and its parental lines were characterized in detail by Stadlmeier et al. [21].

### 4.2. Phenotyping and Phenotypic Data Analysis

The BMWpop was evaluated together with the eight founders and the check variety RGT Reform in ten field trials during 2016–2021. The field trials were conducted at seven locations across Germany: Feldkirchen, Frankendorf, Groß Lüsewitz, Hadmersleben, Morgenrot, Roggenstein, and Söllingen. The experiment was laid out as an alpha lattice with two replications in all environments. The population was grown in plots of 5.3–13.5 m^2^. Field management followed the recommended agricultural practices including the application of fungicides and growth regulators. PH was measured in cm from the ground to the top of the spike after anthesis. HD was defined as the number of days from 1 May until half of the plants showed spikes emerging above the flag leaf ligule. GY was measured in t/ha at 86% dry-matter content. TGW and grain dimensions were assessed using the MARViN seed analyser (MARViTech GmbH, Germany). GPC was determined by near-infrared spectroscopy. Outliers were removed from the raw phenotypic data using an iterative Grubbs outlier test [58,59]. Indices derived from GY and GPC were calculated on a plot basis within each trial. As suggested by Monaghan et al. [9], GPD was defined as the residuals from a linear regression of GPC on GY. Correspondingly, the residuals from a regression of GY on GPC represented GYD. GPY was derived by multiplying GY with GPC. EWPY was defined as the sum of standardised values for GY and GPC. Standardisation was achieved by subtracting the mean and dividing by the standard deviation as described by Rapp et al. [13]. Adjusted entry means for all traits and indices were calculated using a two-stage approach. In the first stage, lattice-adjusted means were estimated for each environment based on the following linear mixed model:

$$y_{ijk}=\mu +g_{i}+r_{j}+b_{jk}+e_{ijk},$$

where *y_ijk_* is the observed plot value, *µ* is the overall mean, *g_i_* is the fixed effect of genotype *i*, *r_j_* is the fixed effect of replication *j*, *b_jk_* is the random effect of the incomplete block *k* nested in replication *j*, and *e_ijk_* is the random residual error of a plot. The repeatability within an environment was estimated as

$$rep^{2}=\frac{\sigma_{G}^{2}}{\sigma_{G}^{2}+\frac{\sigma_{e}^{2}}{2}}.$$

The genotypic variance $\sigma_{G}^{2}$ and the residual variance $\sigma_{e}^{2}$ were estimated using the first-stage model by assuming a random genotypic effect. In the second stage, adjusted means across environments were estimated for all measured traits and indices using a linear mixed model of the form

$$y_{ij}=\mu +g_{i}+t_{j}+e_{ij},\;$$

where *y_ij_* is the adjusted mean from the first stage, *µ* is the overall mean, *g_i_* is the fixed effect of genotype *i*, *t* is the random effect of trial *j*, and *e_ij_* is the random residual. The heritability of a trait was estimated according to the formula:

$$h^{2}=\frac{\sigma_{G}^{2}}{\sigma_{G}^{2}+\frac{\sigma_{e}^{2}}{T}},\;$$

where *T* is the number of trials. Variance components for estimating *h*^2^ were derived from the second-stage model assuming a random genotypic effect. Traits with a repeatability <0.3 in a given environment were excluded from further analysis. Linear mixed models for phenotypic data analysis were fit using the R package lme4 [60,61].

### 4.3. Genotyping and Genotypic Data Analysis

Total genomic DNA was extracted as described by Plaschke et al. [62]. The BMWpop was genotyped by TraitGenetics GmbH, Germany, using an Illumina^®^ iSelect^®^ 20k single-nucleotide polymorphism (SNP) array, which includes a total of 17,267 SNPs from the 90k iSelect array described by Wang et al. [63] and the 820 k Axiom^®^ array reported by Winfield et al. [64]. Physical SNP positions on the RefSeq v1.0 [65] were provided by TraitGenetics GmbH. A genetic map for QTL analysis in the BMWpop was previously published by Stadlmeier et al. [21]. The map comprised 5436 markers distributed over 2804 unique loci (Table S3). 

### 4.4. QTL Analysis

QTL detection was performed by composite interval mapping (CIM) implemented in the R package mpMap [66] using phenotypic data across environments and from individual trials. The probability that an allele was identical by descent with one of the eight founders was calculated for all founders at a grid of 1-centimorgan intervals and at all 2804 unique marker loci. A genome-wide significance threshold for QTL detection was determined by 10,000 permutations of the phenotypic data. The number of cofactors for CIM was set as the number of QTL detected by an initial QTL scan using simple interval mapping. A QTL support interval was defined as the interval in which the −log_10_(*P*) value was within one unit of its maximum. A QTL was only declared significant if it was detected analysing phenotypic means across environments. Effects of founder alleles were estimated in a single-QTL regression model with discrete founder alleles as independent variables. If none of the eight founder probabilities at a given locus reached the threshold of 0.5, the descent of the allele was defined as unknown. The effect of a founder allele was reported as the centred mean of all lines that were identical by descent for the particular allele.

### 4.5. Candidate Genes and Previously Identified QTL

To identify candidate genes for the detected QTL, we focused on genes and gene families that were shown to affect GY parameters or GPC-related traits in previous experiments in wheat and rice. We considered a total of 85 high-confidence genes encoding for proteins such as nitrate transporter, nitrate reductase, nitrite reductase, glutamine synthetase, glutamate dehydrogenase, alanine aminotransferase, and sucrose synthase, among others (Table S4). Candidate genes were searched in the interval spanned by the QTL peak markers ±5 Mb. No candidate genes were searched for QTL that were mapped more than one centimorgan away from the next marker. Gene IDs and physical positions were retrieved from the IWGSC RefSeq v1.0 and v1.1 annotation (https://wheat-urgi.versailles.inra.fr/Seq-Repository/Annotations; accessed on 9 June 2022). Gene descriptions were obtained from the Ensembl Plants database (http://plants.ensembl.org; accessed on 9 June 2022) using the R package biomaRt [67]. To identify colocalizing QTL from previous studies, we compared physical support intervals of the QTL of the present study to those detected in the two meta-QTL analyses by Saini et al. [54] (for GY, HD, PH, and grain morphometric traits) and Gudi et al. [55] (for GPC) and the genome-wide association studies by Nigro et al. [14] and Thorwarth et al. [15] (for GY-GPC indices).

## Figures and Tables

**Table 1:** Summary of phenotypic data for the BMWpop across ten environments: number of environments (Env.) for genetic analysis (filtered for *rep*^2^ ≥ 0.3 for all measured traits), range of repeatability (*rep*^2^), heritability (*h*^2^), genotypic variance (*σ_G_*^2^), environmental variance (*σ_E_*^2^), residual variance (*σ_R_*^2^), range of adjusted means, overall mean and standard deviation (SD).

Trait	Env.	*rep* ^2^	*h* ^2^	*σ_G_* ^2^	*σ_E_* ^2^	*σ_R_* ^2^	Range	Mean ± SD
Grain yield (GY; t/ha)	9	0.71–0.92	0.83	0.17	0.83	0.31	6.2–9.3	8.1 ± 0.5
Grain protein content (GPC; %)	9	0.80–0.93	0.93	0.40	1.11	0.28	11.8–16.2	13.6 ± 0.7
Grain protein deviation (GPD; %)	8	0.67–0.90	0.88	0.21	<0.01	0.22	−1.6–1.6	0.0 ± 0.5
Grain yield deviation (GYD; t/ha)	8	0.55–0.86	0.76	0.08	<0.01	0.19	−1.4–0.7	0.0 ± 0.3
Grain protein yield (GPY; t/ha)	8	0.38–0.83	0.76	<0.01	0.01	<0.01	0.9–1.2	1.1 ± 0.0
Equal weight protein yield (EWPY)	8	0.29–0.83	0.79	0.19	<0.01	0.41	−1.9–1.3	0.0 ± 0.5
Heading date (HD; d)	7	0.84–0.93	0.96	2.38	18.62	0.73	25.6–35.1	30.1 ± 1.6
Plant height (PH; cm)	7	0.78–0.96	0.97	54.70	44.70	10.60	71.8–110.9	92.1 ± 7.5
Thousand-grain weight (TGW; g)	8	0.69–0.97	0.95	9.95	16.43	3.91	34.6–53.7	43.4 ± 3.2
Grain width (GW; mm)	7	0.70–0.95	0.94	0.01	0.02	<0.01	3.1–3.8	3.5 ± 0.1
Grain length (GL; mm)	7	0.81–0.99	0.97	0.05	0.02	0.01	5.6–6.9	6.2 ± 0.2

**Table 2:** Phenotypic correlations of the BMWpop across environments.

	GY	GPC	GPD	GYD	GPY	EWPY	HD	PH	TGW	GW
GPC	−0.60									
GPD	−0.28	0.92								
GYD	0.86	−0.20	0.18							
GPY	0.58	0.24	0.58	0.90						
EWPY	0.27	0.56	0.84	0.69	0.93					
HD	−0.17	ns	−0.10	−0.18	−0.20	−0.19				
PH	ns	0.25	0.20	ns	0.14	0.17	ns			
TGW	0.21	0.22	0.30	0.31	0.39	0.38	−0.20	0.51		
GW	0.13	0.28	0.33	0.26	0.37	0.38	ns	0.46	0.86	
GL	0.26	ns	ns	0.26	0.23	0.16	−0.17	0.25	0.70	0.35

ns: not significantly different from zero at *α* = 0.05.

**Table 3:** Characterization of quantitative trait loci identified by composite interval mapping: chromosome (Chr.), QTL identifier, position on the genetic map with the support interval in centimorgans, number of environments (Env.) in which the QTL was observed, −log_10_(*p*) value, explained phenotypic variance (*R*^2^), and candidate gene. All shown QTL were detected across trials.

Trait	Chr.	QTL	Position	Env.	−log_10_(*p*)	*R* ^2^	Candidate Gene
GY	2A	*QGy.lfl-2A*	139.6 (137.0–143.0)	3	8.0	0.10	-
	4A	*QGy.lfl-4A*	183.6 (176.0–184.6)	3	6.1	0.08	*TaGS3-4A*
	5A	*QGy.lfl-5A*	99.0 (89.1–109.0)	1	5.4	0.06	-
	5B	*QGy.lfl-5B*	126.7 (124.0–137.0)	0	5.2	0.06	-
	6A	*QGy.lfl-6A*	44.5 (40.5–50.0)	0	6.0	0.04	-
	6B	*QGy.lfl-6B*	245.0 (235.1–249.8)	2	5.7	0.04	-
GPC	1A	*QGpc.lfl-1A*	218.0 (212.3–220.2)	1	4.6	<0.01	-
	2A	*QGpc.lfl-2A*	127.3 (119.0–131.0)	6	13.1	0.09	-
	2B	*QGpc.lfl-2B*	271.0 (265.7–277.5)	0	5.2	0.04	-
	2D	*QGpc.lfl-2D*	108.6 (100.0–113.0)	3	7.1	0.07	-
	3A	*QGpc.lfl-3A*	111.0 (98.0–116.1)	1	7.0	0.08	-
	4A	*QGpc.lfl-4A*	139.0 (137.6–144.0)	2	9.5	0.06	-
	4B	*QGpc.lfl-4B*	75.0 (71.0–80.5)	3	13.2	0.10	*Rht-B1*; *TaGSe-4B*
	4D	*QGpc.lfl-4D*	29.0 (17.0–38.0)	4	8.4	0.07	*Rht-D1*
	5A	*QGpc.lfl-5A*	29.0 (24.0–38.0)	0	8.4	0.07	-
	5B	*QGpc.lfl-5B*	302.5 (291.5–302.5)	1	5.9	0.04	-
	6A	*QGpc.lfl-6A*	179.2 (170.0–186.0)	1	8.6	0.07	-
	6B	*QGpc.lfl-6B*	156.1 (154.0–161.0)	2	8.4	0.05	-
GPD	2A	*QGpd.lfl-2A.1*	127.8 (122.0–131.9)	2	9.1	0.08	-
	2A	*QGpd.lfl-2A.2*	230.6 (227.0–234.7)	0	6.6	0.06	-
	3A	*QGpd.lfl-3A*	112.0 (109.9–125.0)	1	5.6	0.08	-
	3B	*QGpd.lfl-3B*	255.9 (254.4–266.0)	1	4.6	<0.01	-
	4D	*QGpd.lfl-4D*	12.0 (0.0–17.0)	1	6.9	0.04	-
	5B	*QGpd.lfl-5B*	61.0 (52.0–63.7)	0	4.7	0.03	-
	7A	*QGpd.lfl-7A.1*	80.4 (78.0–82.5)	1	10.4	0.04	*TaTEF-7A*
	7A	*QGpd.lfl-7A.2*	219.5 (215.0–227.0)	1	6.2	0.05	*NRT2.6-7A*
	7A	*QGpd.lfl-7A.3*	323.5 (322.0–326.0)	2	4.8	0.04	*WAPO-A1*
GYD	1B	*QGyd.lfl-1B*	73.3 (68.0–76.0)	2	5.8	0.08	-
	2A	*QGyd.lfl-2A*	139.0 (134.0–143.0)	0	5.4	0.06	-
	4A	*QGyd.lfl-4A*	159.3 (154.0–161.8)	1	5.3	0.08	TraesCS4A02G446700
GPY	1B	*QGpy.lfl-1B*	67.2 (57.0–75.0)	2	4.9	0.08	-
EWPY	1B	*QEwpy.lfl-1B*	59.4 (52.8–67.7)	1	5.4	0.06	-
	2B	*QEwpy.lfl-2B*	186.8 (182.3–199.0)	1	6.3	0.07	-
	7A	*QEwpy.lfl-7A*	323.5 (323.0–327.0)	0	4.5	0.01	*WAPO-A1*
HD	1B	*QHd.lfl-1B*	333.0 (324.0–345.0)	3	8.1	0.04	-
	3A	*QHd.lfl-3A*	214.5 (209.4–223.0)	1	4.3	0.05	-
	4B	*QHd.lfl-4B*	116.7 (110.0–124.0)	3	7.4	0.06	-
	5B	*QHd.lfl-5B*	103.8 (96.2–105.8)	3	8.2	0.10	-
	6D	*QHd.lfl-6D*	70.0 (63.0–78.0)	3	7.8	0.02	-
	7A	*QHd.lfl-7A*	94.0 (91.2–102.0)	1	8.2	0.09	-
PH	4B	*QPh.lfl-4B*	89.4 (83.0–95.7)	6	13.3	0.11	*Rht-B1*
	4D	*QPh.lfl-4D*	27.4 (23.0–31.0)	7	50.4	0.27	*Rht-D1*
	6A	*QPh.lfl-6A*	159.4 (148.0–161.0)	7	17.4	0.12	*Rht24*
	6D	*QPh.lfl-6D*	68.5 (67.5–94.0)	6	7.5	0.03	-
TGW	2A	*QTgw.lfl-2A*	125.3 (121.8–136.5)	5	8.8	0.05	-
	2B	*QTgw.lfl-2B*	178.8 (168.6–188.9)	6	4.3	0.07	-
	4D	*QTgw.lfl-4D*	25.0 (17.0–38.0)	5	6.7	0.13	*Rht-D1*
	6A	*QTgw.lfl-6A*	130.0 (128.7–134.0)	2	8.3	0.03	-
GW	1A	*QGw.lfl-1A*	155.8 (153.3–165.0)	2	4.5	0.08	-
	2A	*QGw.lfl-2A*	136.0 (126.3–144.7)	2	5.2	0.08	-
	4D	*QGw.lfl-4D*	23.0 (17.0–34.0)	6	12.8	0.20	*Rht-D1*
	6A	*QGw.lfl-6A*	156.0 (145.0–164.0)	2	4.7	0.04	*Rht24*
	6B	*QGw.lfl-6B*	72.9 (60.0–80.0)	3	5.5	0.06	-
GL	1A	*QGl.lfl-1A*	174.9 (164.0–181.0)	3	4.9	0.03	-
	1B	*QGl.lfl-1B.1*	23.0 (20.0–33.6)	6	6.9	0.11	-
	1B	*QGl.lfl-1B.2*	238.9 (235.0–242.0)	5	8.0	0.06	-
	2B	*QGl.lfl-2B*	182.3 (178.8–183.8)	4	10.3	0.04	-
	3A	*QGl.lfl-3A*	239.0 (230.2–241.0)	3	6.3	0.06	-
	3B	*QGl.lfl-3B*	142.9 (139.0–150.0)	4	4.7	0.04	-
	6D	*QGl.lfl-6D*	153.0 (140.0–157.0)	5	10.8	0.06	-
	7A	*QGl.lfl-7A.1*	60.0 (52.5–70.0)	4	7.5	0.10	-
	7A	*QGl.lfl-7A.2*	324.0 (310.0–327.0)	3	5.1	<0.01	*WAPO-A1*

## Data Availability

Not applicable.

## Supplementary Materials

The following Supporting Information can be downloaded at: https://www.mdpi.com/article/10.3390/plants11162146/s1, Figure S1: Distribution of adjusted means in the BMWpop for the traits grain yield (GY), grain protein content (GPC), grain protein deviation (GPD), grain yield deviation (GYD), grain protein yield (GPY), equal-weight protein yield (EWPY), heading date (HD), plant height (PH), thousand-grain weight (TGW), grain width (GW), and grain length (GL); Table S1: QTL detected within and across environments: genetic position, flanking markers, *p*-value, explained variance, maximal effect, and centred effects of eight founder haplotypes. QTL between markers are denoted by “loc”, followed by the position in centimorgans. Some founder effects could not be estimated (NA) because the founder probability did not reach the threshold; Table S2: Interval for the identification of candidate genes and the number of annotated gene models (RefSeq v1.1); Table S3: Linkage map of the BMWpop; Table S4: Genes considered as candidates for the detected QTL.
