# Supplementary material for: Genetics of the Inverse Relationship between Grain Yield and Grain Protein Content in Common Wheat
Source: Plants (Basel). 2022 Aug 18;11(16):2146. doi: 10.3390/plants11162146 (PMC9413592; doi:10.3390/plants11162146)
Supplement: Supplementary file 1 [file plants-11-02146-s001.zip › Figure_S1.pdf]

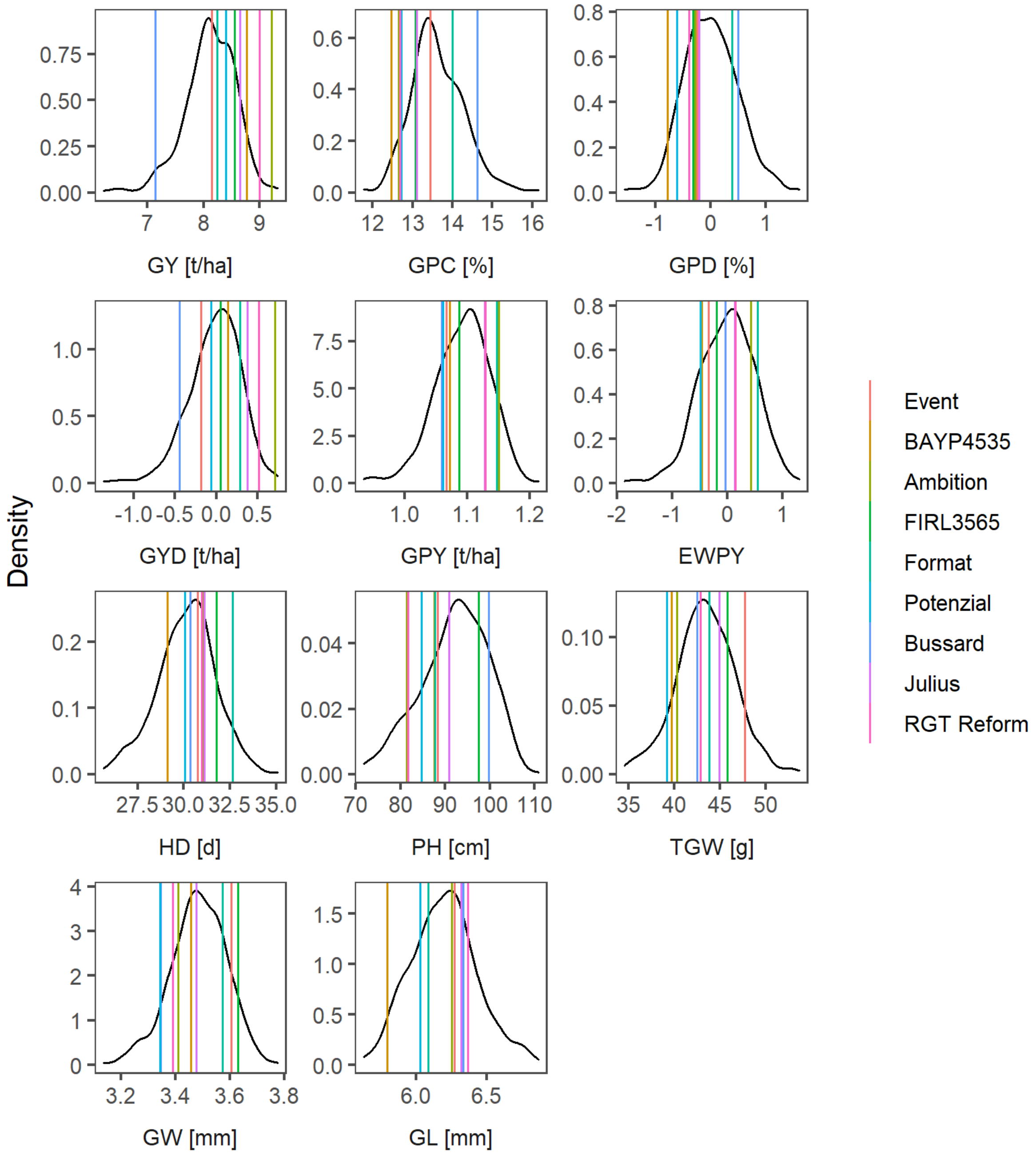

Figure S1: Distribution of adjusted means in the BMWpop for the traits grain yield (GY), grain protein content (GPC), grain protein deviation (GPD), grain yield deviation (GYD), grain protein yield (GPY), equal-weight protein yield (EWPY), heading date (HD), plant height (PH), thousand-grain weight (TGW), grain width (GW) and grain length (GL).
